# Neuropathies related to hepatitis E virus infection: First reported case in Europe

**DOI:** 10.1111/ene.16136

**Published:** 2023-11-09

**Authors:** Joaquín Bravo Urbieta, Miguel Martín Cascón, Sergio Alemán Belando

**Affiliations:** ^1^ Infectious Diseases Unit, Internal Medicine Department Hospital General Universitario José María Morales Meseguer Murcia Region of Murcia Spain; ^2^ Department of Internal Medicine, Faculty of Medicine University of Murcia El Palmar Region of Murcia Spain; ^3^ Internal Medicine Department Hospital General Universitario José María Morales Meseguer Murcia Region of Murcia Spain

Dear Editor:

We have read the paper by Ripellino et al. [1] and wish to highlight two significant points. First, we would like to draw your attention to the case of peripheral facial paralysis as a complication of acute hepatitis E, a description of which we recently published in this very journal. This represents the first reported case in Europe [2]. Second, regarding the publication itself, it is stated that no association has been found between hepatitis E and peripheral facial paralysis, leading to the conclusion that routine testing for hepatitis E for clinical purposes is not recommended. However, we believe caution is warranted in making this assertion for several reasons.

Firstly, it is important to note that the absence of evidence does not equate to evidence of the absence of a relationship between the two conditions. As noted in the discussion of the study's limitations, the sample size was small and lacked the necessary statistical power to detect an association with such a rare complication as that under investigation. In Europe, one case of hepatitis E is reported for every 3109 blood donations [3]. Given the low incidence of facial paralysis in relation to acute hepatitis E, a considerably larger number of cases and corresponding controls would be required to detect a statistically significant difference than the 70 cases reported in the study in question.

Secondly, peripheral facial paralysis is a clinical syndrome with many potential etiologies. Infectious causes are not uncommon, and in some cases, serological diagnosis is imperative [4]. Obtaining a serum sample allows for the diagnosis of various conditions, such as Lyme disease or HIV infection, among other infections, and even for further investigations, such as viral load determination from the same sample. We believe that the cost of incorporating serological screening for hepatitis E virus is relatively low in the context of comprehensive patient care. Considering these factors, the European Association for the Study of the Liver guidelines recommend serological screening for hepatitis E in patients with extrahepatic manifestations, even when transaminase levels are normal [5]. In the cases referred to in this study, immunogloblulin M determination was positive in all cases, and in the case of our patient, the presence of viremia was also demonstrated.

Thirdly, the neurotropic potential of hepatitis E virus is well documented [6]. The limited number of published cases likely reflects a rare complication, but it does not rule out its existence.

Finally, it is crucial to note that there is a specific antiviral treatment for hepatitis E: ribavirin. Therefore, the confirmation of acute hepatitis E could have implications for the possibility of administering this treatment, even though there is not yet sufficient evidence to support its use in patients with neurological manifestations related to hepatitis E.

In summary, we recommend the inclusion of serological screening for hepatitis E in the initial evaluation of patients with peripheral facial paralysis, as recommended by clinical practice guidelines.

## AUTHOR CONTRIBUTIONS

**Joaquín Bravo Urbieta:** Conceptualization; formal analysis; writing – original draft; writing – review and editing; validation. **Miguel Martín Cascón:** Conceptualization; validation; formal analysis; supervision; writing – review and editing. **Sergio Alemán Belando:** Conceptualization; validation; formal analysis; supervision; writing – review and editing.

## CONFLICT OF INTEREST STATEMENT

The authors declare that they have no conflicts of interest related to this work.

## Data Availability

No data are available for sharing, no Data Availability Statement is provided.
